# A biochemical, theoretical and immunohistochemical study comparing the therapeutic efficacy of curcumin and taurine on T-2 toxin induced hepatotoxicity in rats

**DOI:** 10.3389/fmolb.2023.1172403

**Published:** 2023-05-04

**Authors:** Maryam H. Al-Zahrani, Maha J. Balgoon, Nagwa M. El-Sawi, Fawzia A. Alshubaily, Ebtihaj J. Jambi, Sohair M. Khojah, Raghad S. Baljoon, Nuha A. Alkhattabi, Lina A. Baz, Asmaa A. Alharbi, Amira M. Ahmed, Ayat M. Abo elkhair, Mohamed Ismael, Sahar M. Gebril

**Affiliations:** ^1^ Biochemistry Department, Faculty of Science, King Abdulaziz University, Jeddah, Saudi Arabia; ^2^ Department of Chemistry, Faculty of Science, Sohag University, Sohag, Egypt; ^3^ Ibn Sina National College for Medical Studies, Jeddah, Saudi Arabia; ^4^ Biochemistry Department, Faculty of Pharmacy, Beni Suef University, Beni Suef, Egypt; ^5^ Histology and Cell biology Department, Faculty of Medicine, Sohag University, Sohag, Egypt

**Keywords:** T-2 toxin, hepatotoxicity, fibrosis, DNA damage, apoptosis, curcumin, taurine, molecular docking

## Abstract

**Introduction:** Foodborne trichothecene T-2 Toxin, is a highly toxic metabolite produced by Fusarium species contaminating animal and human food, causing multiple organ failure and health hazards. T-2 toxins induce hepatotoxicity via oxidative stress causing hepatocytes cytotoxicity and genotoxicity. In this study, curcumin and taurine were investigated and compared as antioxidants against T-2-provoked hepatotoxicity.

**Methods:** Wistar rats were administrated T-2 toxin sublethal oral dose (0.1 mg/kg) for 2 months, followed by curcumin (80 mg/kg) and taurine (50 mg/kg) for 3 weeks. Biochemical assessment of liver enzymes, lipid profiles, thiobarbituric acid reactive substances (TBARs), AFU, TNF-α, total glutathione, molecular docking, histological and immunohistochemical markers for anti-transforming growth factor-β1 (TGFβ1), double-strand DNA damage (H2AX), regeneration (KI67) and apoptosis (Active caspase3) were done.

**Results and Discussion:** Compared to T-2 toxin, curcumin and taurine treatment significantly ameliorated hepatoxicity as; hemoglobin, hematocrit and glutathione, hepatic glycogen, and KI-67 immune-reactive hepatocytes were significantly increased. Although, liver enzymes, inflammation, fibrosis, TGFβ1 immunoexpressing and H2AX and active caspase 3 positive hepatocytes were significantly decreased. Noteworthy, curcumin’s therapeutic effect was superior to taurine by histomorphometry parameters. Furthermore, molecular docking of the structural influence of curcumin and taurine on the DNA sequence showed curcumin’s higher binding affinity than taurine.

**Conclusion:** Both curcumin and taurine ameliorated T-2 induced hepatotoxicity as strong antioxidative agents with more effectiveness for curcumin.

## 1 Introduction

Trichothecene (TCT) mycotoxins are chemically related compounds produced by more than one genus of fungi. T-2 toxin has intense lethality and the highest toxicity compared to TCT other members worldwide and represents a great threat to humans and animals, causing massive financial burden (Milićević et al., 2010; Nayakwadi et al., 2020; Zhang et al., 2022a). Mycotoxins are a specialized group of low molecular weight organic secondary metabolites of pathological microfungi related to Penicillium, Fusarium, and Aspergillus categories (Zhang et al., 2022b).

Naturally occurring as 12,13-epoxytrichothecene mycotoxin, T-2 toxin is a non-volatile compound with a low molecular weight that is dissolvable in water and petroleum ether. It is, however, very soluble in acetone, ethyl-acetate, dimethyl soapboxed, chloroform, ethyl alcohol, methyl alcohol, and propylene glycol (Janik et al., 2021; Polak-Śliwińska and Paszczyk, 2021).

In addition, it is known for its ability to withstand heat and UV light, making food production, food processing, or even autoclaving unable to inactivate it (Afsah-Hejri et al., 2013). A study showed that T-2 toxin could be inactivated by heating at 200°C–210°C for 30–40 min or soaking in sodium hypochlorite-sodium hydroxide solution for at least 4 hours (Hesketh et al., 1991; Li et al., 2011). Despite the great effort to find physical and chemical approaches to get rid of mycotoxins, some limitations are present, like safety issues, important nutritive value loss, and tremendous cost. These limitations still hinder their food industrial application (Huang et al., 2018).

T-2 toxicity varies depending on the route of infection and, therefore, can range from acute to chronic, causing apoptosis in the immune system and fetal tissues after ingestion. T-2 toxin is typically metabolized and eliminated after ingestion, producing over 20 metabolites (Zhang et al., 2022a). As a result, human consumption of animal products may be tainted with T-2 toxin and its metabolites leading to various organ toxicity, including liver, kidney, skin, and reproductive organs (Janik et al., 2021). T-2 toxins, with their lipophilic nature, can be absorbed from the alimentary tract and the respiratory mucosal membranes. However, the liver is the most affected organ as it is the primary site for its metabolism and elimination after absorption (Yang et al., 2013). T-2 toxin has been shown to damage multiple tissues and cells under oxidative stress in both *in vitro* and *in vivo* studies (Zhang et al., 2018; Yin et al., 2020; Yang et al., 2021).

Curcumin, a polyphenolic curcuminoid, is among the most exhaustively studied active natural products and the most used curcuminoid of turmeric. It has been proven as the primary effector in various for its biological activities as anticancer, antioxidant, anti-inflammatory, antimutagenic, antimicrobial, and antimutagenic properties (Jyotirmayee and Mahalik, 2022). In some studies, curcumin has also been shown to reduce AFB1 toxicity by increasing antioxidant enzyme activity, neutralizing free radicals, and preventing AFB1 bioconversion into its 8, 9-epoxide version (Limaye et al., 2018; Pauletto et al., 2020). Despite the demonstrated positive effects of curcumin, its beneficial effects were indeed limited by its low solubility and poor bioavailability (Anand et al., 2007; Liu et al., 2016), prompting researchers to employ a variety of methods to improve curcumin solubility and bioavailability (Hernandez-Patlan et al., 2019; Pan-On et al., 2022).

Taurine, 2-aminoethanesulfonic acid, is an amino acid derivative that was for the first time extracted from the bile of ox in 1927. Also, we can obtain it from diet or synthesize *de novo* from certain amino acid catabolism (Russell, 2003). Taurine as a supplement has been widely used due to its beneficial role in many diseases, such as hypertension (Sun et al., 2016). Taurine acts as a natural antioxidant and stress adaptor and has become under research focus during the last three decades with an antitumor activity that can scavenge oxygen free radicals (Surai et al., 2021).

Additionally, taurine proved its efficacy in cellular protection against protective cells against the cytotoxicity associated with oxidative stress acting as an anti-inflammatory (Marcinkiewicz and Kontny, 2014). Previous literature has proved the physiological and cellular role of taurine in suppressing oxidative stress, inflammatory response, and specific cytochrome isozymes and in conjugating the bile acids with xenobiotics (Nikkhah et al., 2021) and against various hepato-toxic agents (Abdel-Daim et al., 2019; Elwy et al., 2019). Also, it plays a vital role in cellular membranes stabilization cell membranes, controls glutathione reserve in the liver, anti-apoptotic, osmoregulation and neurons excitability (Rafiee et al., 2022). Moreover, using taurine in specific amounts decreased tumor growth rate with the extension of the mean lifespan (Yu and Kim, 2010).

The aim of this study is to evaluate how effective curcumin and taurine are at reducing the hepatotoxic effects of T-2 toxin on liver performance and morphology. Serum biochemical assessment of liver enzymes, lipid profile, inflammatory and antioxidant parameters, liver histopathological and immunohistochemical parameters were investigated.

## 2 Materials and methods

### 2.1 Ethical approval statement

The whole experimental work was performed following the HELSENKI guidelines. The study was conducted at the Faculty of Medicine, animal facility Sohag University, Sohag, Egypt, according to the guidelines of the Medical Research Ethics Committee (MREC) for experimental animals’ welfare and use approval under IRB registration number: Sohag - IACUC-5-11-2022-1.

### 2.2 Chemicals

T-2 toxin was dissolved in propylene glycol and was adjusted to be 0.1 mg/kg body weight). Curcumin and Taurine were purchased and diluted in distal water to be used by (80 mg/kg body weight/day) (50 mg/kg) respectively, to be given by gastric gavage daily for 3 weeks. All chemicals were purchased from Sigma-Aldrich Chemical Company, Missouri, United States.

### 2.3 Animals and experimental design

Forty healthy adult male Wistar rats weighing (210–240 g) were obtained from the National Research Centre (NRC), Cairo, Egypt. Rats were housed for 1 week in the experimental room before starting the experiments to achieve acclimatization conditions. Metal cages were used to keep them safe, considering hygienic conditions; temperature was 25°C and humidity 55% through a 12-light/12-dark cycle, with access to standard rat standard pellet food *ad libitum*. Then, the rats were equally and indiscriminately divided into four groups: (*N* = 10). as follows:

Group C (control group): Rats were given propylene glycol (0.5 mL/kg/day) injected intraperitoneally once daily for 2 months and considered a negative control.

Group T1 (T-toxin group): Rats were T-2 toxin (0.1 mg/kg) dissolved propylene glycol once daily injected intraperitoneally for 2 months (El-Sawi and Al-Seeni, 2009).

Group T2 (Curcumin treated group): after administration of T-2 toxin for 2 months, rats were treated with curcumin (80 mg/kg) dissolved in distilled water once daily by gastric gavage for 3 weeks (Labban et al., 2021).

GroupT3 (Taurine treated group): after administration of T-2 toxin for 2 months, rats were treated with taurine (50 mg/kg) dissolved in distilled water once daily by gastric gavage for 3 weeks (Mousavi et al., 2020).

At the end of the experiment duration, 1 day following the last dose with fasting overnight, anesthetized rats with a combination of 50 mg/kg ketamine and 5 mg/kg xylazine (Koc et al., 2005). Retro-orbital blood samples were collected using capillary tubes and centrifuged for 15 min at 3,000 rpm. Separated serum was aliquoted and stored in Eppendorf tubes at −20°C for further biochemical analysis. Liver tissue was dissected, washed in saline, trimmed into small strips and fixed in 10% neutral buffered formalin for 48 h Before processing for histological and immunohistochemical assessment and evaluation.

### 2.4 Biochemical assessment

#### 2.4.1 Evaluation of liver enzymes

Blood serum aliquots were analyzed by Eliza kits for Alanine Aminotransferase (ALT) (Cat No: E4325-100) and Aspartate aminotransferase (AST) (Cat. No: E4321-100), Alkaline phosphatase (ALP) (Cat. No: E45751-100) obtained by Bio vision (California, USA). All tests were done following the manufacturer’s data sheet instructions.

#### 2.4.2 Estimation of total lipid, total cholesterol, lipid peroxidation indicator parameter (TBARs), total and reduced GSH expression levels

Serum estimation of reduced glutathione (GSH) (cat. No. MBS724319, California, United States). Serum estimation of total cholesterol (cat. No. CH 12 20, Biodiagnostic). Serum estimation of lipid peroxide (cat. No. MD 25 29, Biodiagnostic). All tests were done following the manufacturer’s data sheet instructions.

#### 2.4.3 Detection and evaluation alpha-L-fucosidase (AFU) and tumor necrosis factor-alpha (TNF-α); tumor markers

ELISA kits for rats were used to estimate the corresponding tumor markers in the liver tissue homogenate. Rat Alpha-L-Fructosidase (AFU) Cat. No E0086Ra) and tumor necrosis factor-alpha (TNF-α) (Cat. No E0764Ra) BT -LAB Eliza kits were obtained from (Shanghai, China). All steps and protocols were done following the manufacturer’s data sheet instructions.

#### 2.4.4 Assessment of hematological parameters (hemoglobin (Hb), hematocrit (HC)

The biochemical tests included the determination of hemoglobin was made using the oxyhemoglobin method (Attia et al., 2015), hematocrit (Malenica et al., 2017), and glutathione (Francioso et al., 2021), thiobarbituric acid reactive substances (TBAR; 26), total glutathione (Zeb and Ullah, 2016), total lipids (Shapaval et al., 2019) and total serum cholesterol (Valdivia and Bhattacharya, 2022) were measured.

### 2.5 Histopathological examinations

#### 2.5.1 Microscopic evaluation of liver tissue by general and special histological stains

The histological evaluation was done at the histology department, Faculty of Medicine, Sohag University. Liver tissue strips at 5 × 10 mm in diameter were fixed using 10% neutral buffered formalin for 48 h s. Fixed samples were washed and processed automatically in the tissue processor (Leica TP1020 Semi-enclosed Benchtop Tissue Processor GmbH, D-69226 Nussloch, Germany) embedded in paraffin blocks. Paraffin tissue was cut at 4 µm sections using a rotatory microtome and then mounted over glass slides for all groups samples. Four µm liver sections were processed and stained generally by hematoxylin and eosin (H&E) stain for histopathological changes evaluation, specifically by Periodic acid Schiff (PAS) for glycogen content of hepatocytes (Hui et al., 2017), Sirius red and Masson trichrome for collagen fibers demonstration (Huang et al., 2013), the technique for processing. Staining was done according to the method of (Horobin, 2013). All the stained sections were examined by Olympus microscope model BX51 and the photos were captured using an Olympus DP25 camera.

#### 2.5.2 Immunohistochemical staining of anti -KI-67, TGF-β1, phospho histone H2AX (H2AX) and active caspase 3

Procedures were processed according to the products manufacturer’ instructions and other studies (Gebril et al., 2020; Abbas et al., 2022). Four-μm-liver tissue sections were deparaffinized, rehydrated, and subjected to 10 mM sodium citrate buffer antigen retrieval (pH 6.0) for two consecutive runs in a microwave each for 4 min then left to cool down at room temperature. Furthermore, sections underwent endogenous peroxidase blocking for 10 min in hydrogen peroxide (3%), non-specific reactions were stopped by 10% normal goat serum. Primary antibody incubation at 4°C overnight (anti KI-67 (1:300), TGF-β1 (1:150), H2AX (1:500) and active caspase 3 (1:50), then anti-polyvalent and HRP secondary antibodies were applied. DAB chromogen immunodetection was done using (3, 30-diaminobenzidine tetrahydrochloride), Mayer’s hematoxylin is used as the final counterstain step. The primary antibody was removed and replaced with PBS as a negative control. The primary antibody was removed and replaced with PBS as a negative control. Under the same settings, images were taken of immuno-stained sections using a Leica DFC310 FX 1.4-megapixel digital color camera with the Leica software suite LAS EZ V 3.4.0, Leica Microsystems, Germany. Under the same setting conditions. Anti-KI 67 (Cat. No: GB111499) andH2AX (Cat. No: GB111508) from Servicebio Technology Co., Ltd., Wuhan, China. Anti (TGF-β1) (Cat. No: A15103) from ABclonal, Wuhan, China. Anti-active caspase3(Cat. No: 1-CA220-02) from quartett, Berlin, Germany. Immunohistochemistry (DAB) Detection Kits HRP Anti-Polyvalent (AMF080-IFU), ScyTek Laboratories, Inc, United States

#### 2.5.3 Histomorphometry

Different parameters obtained from liver sections stained by, histochemical and immunohistochemical evaluation were statistically analyzed: collagen fibers surface percentage area around the central vein, porta area and in perisinusoidal space of Disse were estimated. Also, for each group, PAS-positive stained hepatocytes surface area was assessed in 21 non-overlapping fields at ×200Magnification. Furthermore, the number of KI 67, anti-phospho-histone H2AX and active caspase 3 positive hepatocytes and the surface immunoreactivity area percentage for TFG-β1 were measured at ×400 Magnification 21 non-overlying fields for each group. All data measurements were carried out by an Image J analyzer software (Image J/FIJI 1.46r) in all groups.

### 2.6 Molecular modeling

Molecular modeling calculations were performed using Gaussian 09w (Frisch et al., 2008), and UCSF Dock6 programs (Allen et al., 2015). Optimized structures and docking analysis were visualized with Gauss View 5.0 (Dennington et al., 2009) and UCSF Chimera 1.14 (Pettersen et al., 2004), respectively.

### 2.7 Statistical analysis

All parameters were statistically analyzed using GraphPad Prism^®^, statistical analysis software package Version 5.00 for Windows (California, United States). One-way analysis of variables (ANOVA) with Newman-keuls test. All results were expressed as Mean ± Standard deviation (SD) considering the significant probability value less (*p*-value) (*p* < 0.05) between groups.

## 3 Results

### 3.1 Biochemical results

#### 3.1.1 Liver enzymes results

Alanine transaminase (ALT) showed a significant increase in the T1 group compared to C group. Groups T2 and T3 showed a significant decrease in the serum level of enzyme with a percentage of change of 16.2% compared to T1 group (Table 1). Similarly, aspartate transaminase (AST) recorded a significant increase in the T1 group followed by high decline in groups T2 and T3 with percentages of change of 32.2% and 24.2%, respectively (Table 1). Alkaline phosphatase (ALP) also behaved the same with an increase in the T1 group followed by a significant decrease in the T2 group (percentage of change 57.4%) and T3 group (percentage of change 54.3%) (Table 1).

**TABLE 1:** Serum levels of Liver enzymes in rats given T-2 toxin (0.1 mg/kg) after 2 months and treated with curcumin or taurine.

Liver Enzyme (U/L)	Group					
	Control	T-2 toxin	T-2 curcumin treated		T-2 taurine treated	
			Results	Change (%)	Results	Change (%)
ALT	19.76 ± 0.425	31.75±	24.21 ± 0.167***b	−16.2	26.61±	−16.2
		0.646***a			0.151***c	
AST	57.14 ± 0.338	85.41±	57.88±	−32.2	64.75±	−24.2
		0.234***a	0.247 ***b		0.8295***c	
ALP	84.29 ± 0.328	201.3 ± 0.615***a	85.85 ± 0.332***b	−57.4	92.01±	−54.3
					0.4793***c	

Data are expressed as mean ± SE, where the number of an asterisk (*) indicates the levels of significance at *p* < 0.05, 0.01, 0.0001.

Letter a referred to the significant difference between T-2 toxin and control.

Letter b referred to the significant difference between curcumin and T-2 toxin.

Letter c referred to the significant difference between taurine and T-2 toxin.

Change % between curcumin, taurine and T-2 toxin.

#### 3.1.2 Total lipid, total cholesterol, lipid peroxidation indicator parameter (TBARs), total and reduced GSH

According to the results shown in (Table 2) a significant increase of total lipid level in the T-2 toxin group was observed. At the same time, curcumin and taurine treated groups showed a significant decrease with percentage of change 30.4% and 22.6%, respectively. Similar effect was shown for total cholesterol, thiobarbituric acid reactive substances (TBARS) and reduced glutathione (GSH) where T-2 toxin showed a significant upregulation compared to control while curcumin and taurine showed a significant downregulation compared to T-2 toxin with more percentage change in curcumin treated groups than with taurine. On the other hand, total GSH showed a different pattern; while T-2 toxin showed significant increase compared to control, curcumin exhibited significant increase compared to T-2toxin (percentage of change 45.7%) and taurine also exhibited significant increase with lower percentage of change (18.6%) than curcumin.

**TABLE 2:** Serum levels of Total lipid, Total cholesterol, lipid peroxidation indicator parameter (TBARs), Total glutathione, reduced GSH in rats given T-2 toxin (0.1 mg/kg) after 2 months then treated with curcumin or taurine.

Parameter	Group					
	Control	T-2 toxin	T-2 curcumin treated		T-2 taurine treated	
			Results	Change (%)	Results	Change (%)
Total lipid (mg/mL)	2.714±	3.887±	2.706±	−30.4	3.007±	−22.6
	0.078	0.055***a	0.008 ***b		0.003***c	
Total cholesterol (mmol/L)	2.013±	3.594 ±	2.092±	−41.8	2.378±	−33.8
	0.002	0.135***a	0.037 ***b		0.073***c	
TBARs (µmol/mL)	15.03±	20.13±	15.38±	−23.6	16.22±	−19.4
	0.021	0.137 ***a	0.152 ***b		0.298 ***c	
Total GSH (mmol/gm wet weight)	0.795±	1.285±	1.845±	45.7	1.523±	18.6
	0.0228	0.057***a	0.0343***b		0.0543***c	
GSH (mmol/mL)	0.932±	1.967±	0.914±	−53.5	0.996±	−49.4
	0.026	0.012***a	0.007***b		0.001***c	

Data are expressed as mean ± SE, where the number of asterisks (*) indicates the levels of significant at *p* < 0.05, 0.01, 0.0001.

Letter a referred to the significant difference between T-2 toxin and control.

Letter b referred to the significant difference between curcumin and T-2 toxin.

Letter c referred to the significant difference between taurine and T-2 toxin.

Change % between curcumin, taurine and T-2 toxin.

#### 3.1.3 Tumor markers results

Alpha-L-fucosidase (AFU), as one of the measured tumor markers in this protocol, exhibited highly significant increase in T-2 toxin group in comparison to control while curcumin and taurine treated groups exhibited significant decrease compared to T-2 group (percentage of change 53.4% and 13.6%, respectively). Like AFU, tumor necrosis factor alpha (TNF-α) recorded highly significant increase in T-2 toxin group in comparison to control and exhibited significant decrease in curcumin treated group compared to T-2 toxin group with percentage of change 37.3% and decrease in taurine treated group with 17.1% percentage of change (Table 3).

**TABLE 3:** Serum level of tumor markers (AFU and TNF-α) of rats given T-2 toxin (0.1 mg/kg) after 2 months and treated with curcumin and taurine.

	Group						
Parameter	Control	T-2 toxin	T-2 +Curcumin treated			T-2+ taurine treated	
			Results		Chang%	Results	Change%
AFU (U/L)	1.041 ± 0.007	8.093 ± 0.01 ***a	3.773 ± 0.04 ***b	−53.4%		6.995 ± 0.19 ***c	−13.6%
TNF-α (pg/mL)	121.4 ± 1.108	493.8 ± 2.676 ***a	309.8 ± 3.769 ***b		−37.3%	409.4 ± 3.74 ***c	−17.1%

Data are expressed as mean ± SE, where the number of asterisks (*) indicates the levels of significance at *p* < 0.05, 0.01, 0.0001.

Letter a referred to the significant difference between T-2 toxin and control.

Letter b referred to the significant difference between curcumin and T-2 toxin.

Letter c referred to the significant difference between taurine and T-2 toxin.

Change % between curcumin, taurine and T-2 toxin.

#### 3.1.4 Hematological parameters results

According to the experimental data showed in (Table 4) Hemoglobin (Hb) measurement showed a significant decrease in T-2 toxin group compared to the control. However, curcumin and taurine treated groups showed significant increase in hemoglobin level compared to T-2 toxin group with percentage of change14% and 5%. Respectively. Similarly, Hematocrit exhibited a significant decrease in group T-2 toxin compared to control group and a significant increase in curcumin and taurine treated groups compared to T2 toxin group with a percentage of change 20.9% and 10.4% respectively.

**TABLE 4:** Levels of Hemoglobin (Hb), Hematocrit in blood of rats given T-2 toxin (0.1 mg/kg) after 2 months and treated with curcumin and taurine.

	Group					
Parameter	Control	T-2 toxin	T-2 curcumin treated		T-2 taurine treated	
			Results	Change% (%)	Results	Chang (%)
Hb **(**gm/100 mL)	8.232 ± 0.120	6.614 ± 0.097 ***a	7.539 ± 0.2 ***b	14	6.943 ± 0.56 *c	5
Hematocrit (%)	54.15 ± 0.641	36.53 ± 0.221 ***a	44.18 ± 0.5 ***b	20.9	40.32 ± 0.446 ***c	10.4

Data are expressed as mean ± SE, where the number of asterisks (*) indicates the levels of significance at *p* < 0.05, 0.01, 0.0001.

Letter a referred to the significant difference between T-2 toxin and control.

Letter b referred to the significant difference between curcumin and T-2 toxin.

Letter c referred to the significant difference between taurine and T-2 toxin.

Change % between curcumin, taurine and T-2 toxin.

According to the experimental data showed in (Table 4) Hemoglobin (Hb) measurement showed significant decrease in T-2 toxin group compared to control. However, curcumin and taurine treated groups showed a significant increase in hemoglobin level compared to T-2 toxin group with a percentage of change14% and 5%. Respectively. Similarly, Hematocrit exhibited a significant decrease in group T-2 toxin compared to control group and a significant increase in curcumin and taurine treated groups compared to T2 toxin group with percentage of change 20.9% and 10.4% respectively.

### 3.2 Histological examination results

#### 3.2.1 General histopathological liver assessment results

**FIGURE 1:**
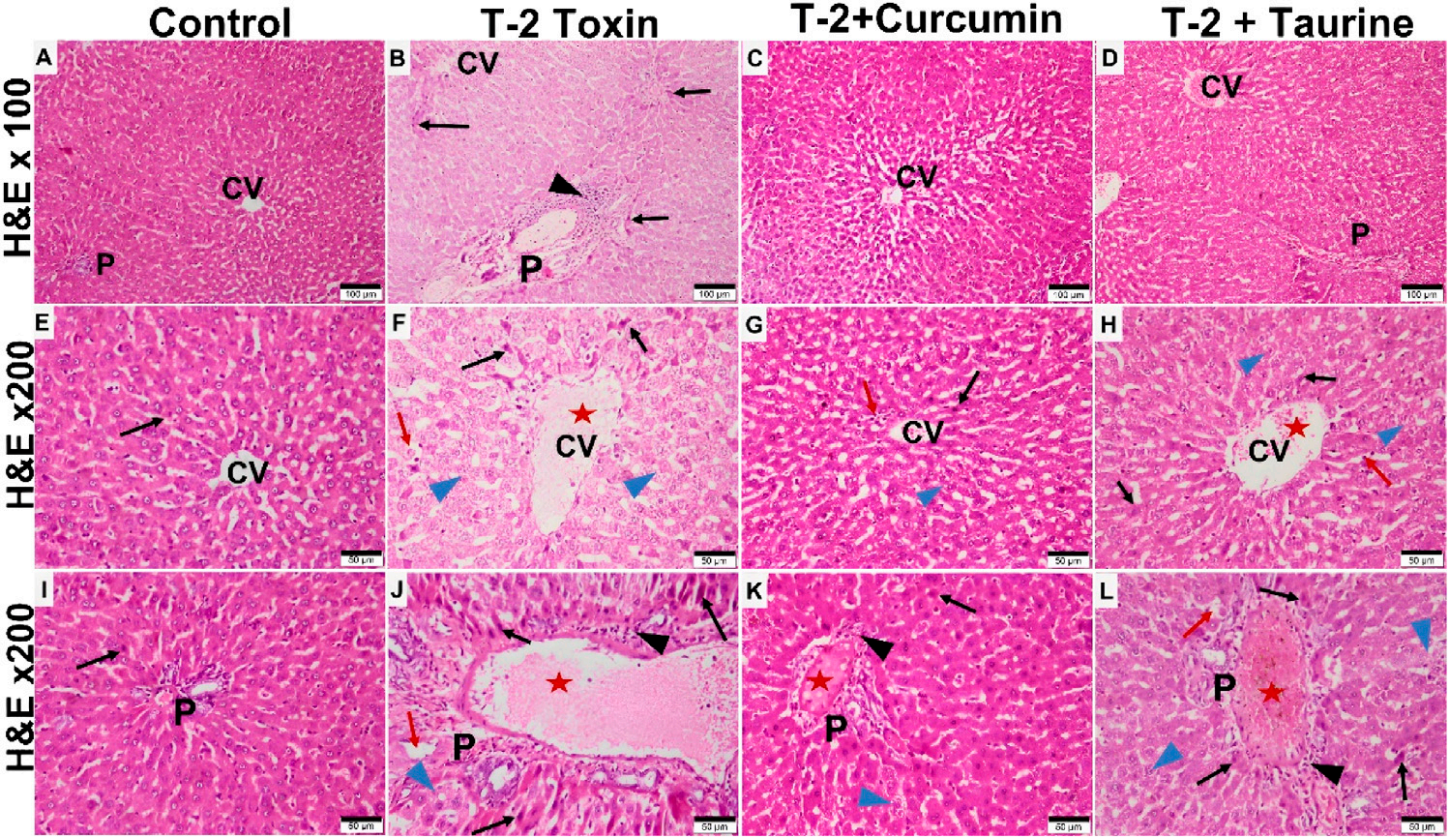
Photomicrographs of H&E-stained liver sections of different groups showing: Control group showing normal liver tissue with classic hepatic lobule formed of central vein (CV) surrounded by portal areas (P) at the periphery. Hepatocytes appear eosinophilic with vesicular nuclei, radiating from the central vein as plates separated by blood sinusoids **(A,E,I)**. T-2 toxin group showing loss of normal hepatic structures, dilated congested CV and portal area (P) (red aster) blood sinusoids (red arrow) with inflammatory cellular infiltration (black arrowhead). Many hepatocytes appear vacuolated and degenerated (blue arrowhead) or dense pyknotic apoptotic nuclei with more eosinophilic cytoplasm (black arrow). **(B,F,J)**. Toxin treated with curcumin groups showing almost normal morphology restoration except for very few cellular infiltration (black arrowhead). Few hepatocytes appear vacuolated (blue arrowhead) or dense pyknotic apoptotic (black arrow) **(C,G,K)**. Toxin treated with taurine group showing restoration of liver tissue but still some congestion in the central vein and the blood sinusoids (red aster). Some hepatocytes still appear vacuolated (blue arrowhead) or with dense pyknotic apoptotic (black arrow) **(D,H,L)** (H & E section at magnification ×100 **(A–D)** with scale bar; 100 µm and ×200 **(E–L)** with scale bar; 50 µm).

Light microscopy examination of H&E-stained liver sections of all groups (Figure 1) evaluation was as follows: control groups of rats showed normal hepatic architecture in the form of cords of hepatocytes radiating from the central vein peripherally toward the normal portal areas. Hepatocytes appear hexagonally with vesicular nuclei in cords separated by normal blood sinusoids. Liver sections of T-2 toxin group showed loss of hepatic architecture, marked hepatocytes vacuolations. Some hepatocytes appear smaller and more eosinophilic with small dense nuclei. Also, the dilation and congestion of the blood vessels including the central vein and portal area were observed with increased Kupffer cells in sinusoids and marked inflammatory cellular infiltration. T-2 toxin rats treated with curcumin showed amelioration and almost normal restoration of the hepatic architecture with mild vascular congestion, few inflammatory cellular infiltrations, and few eosinophilic hepatocytes with small dense nuclei (apoptotic cells). However, the toxic group treated with taurine showed moderate restoration of hepatic architecture except for vascular congestion and moderate inflammatory cellular infiltration mainly at portal area with the presence of some vacuolated (degenerated) hepatocytes and others with eosinophilic pyknotic nuclei (apoptotic cells). Interestingly the ameliorative effect of curcumin in liver tissue hepatoxicity reversibility was greater than with taurine.

#### 3.2.2 Histochemical examination of liver tissue collagen deposition and glycogen content results

**FIGURE 2:**
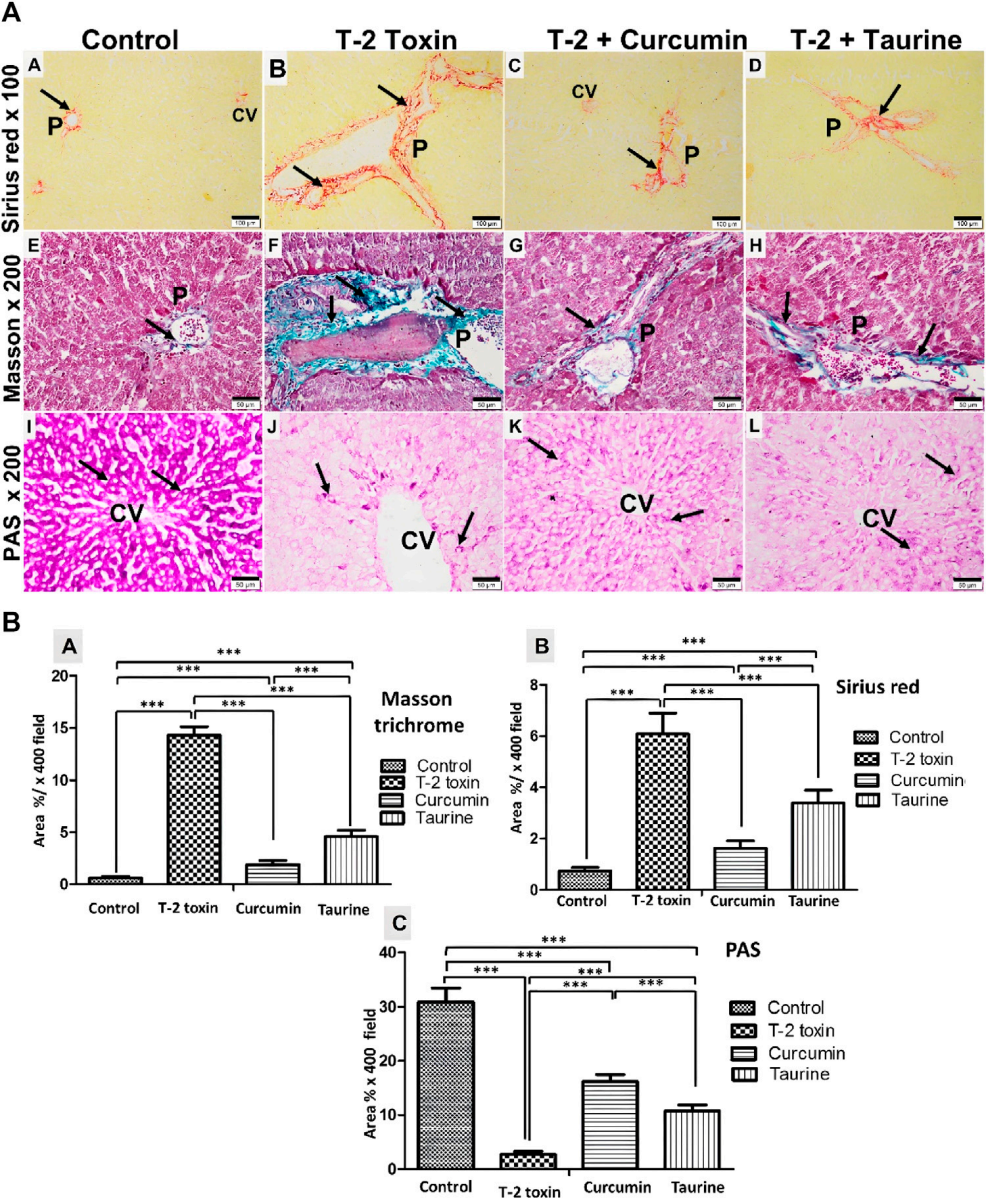
**(A)** Collected photomicrographs of liver sections stained by different histochemical stains, Sirius red, Masson trichrome, and PAS of different groups. Sirius red and Masson trichrome stained control group showing few collagen fibers deposited around the central vein and portal area (A,E). T-2 toxin group showing massive collagen fibrosis mainly around the portal area (B,F). Toxin treated with curcumin group showing group showed a marked decrease in collagen deposits around the central vein and portal area (C,G). Toxin treated with taurine group showing a moderate decrease in collagen fibers around the central vein and portal area (D,H). Collagen fibrosis was demonstrated as red and green color by Sirius red and Masson trichrome, respectively, in all groups. The magenta red of the PAS reaction appears to be a normal concentration around the central vein (CV) in control (I). However, it is depleted in the T-2 toxin group (J). PAS magenta red color reappeared stronger in toxin treated with curcumin group (K) than in toxin treated with taurine group (L). (Sirius red at x 100 (A–D) with scale bar; 100 µm. Masson trichrome and PAS magnification at ×200 (E–L) with scale bar; 50 µm). **(B)** Collected Graphs for histochemical morphometry statistical analysis of Area percentage of Masson trichrome (A), Sirius red (B) for collagen fibrosis and (PAS) for glycogen storage (C). Data are expressed as mean ± SD. ***P = significant difference less than 0.05, analysis of different liver tissue sections from all other groups.

**FIGURE 3:**
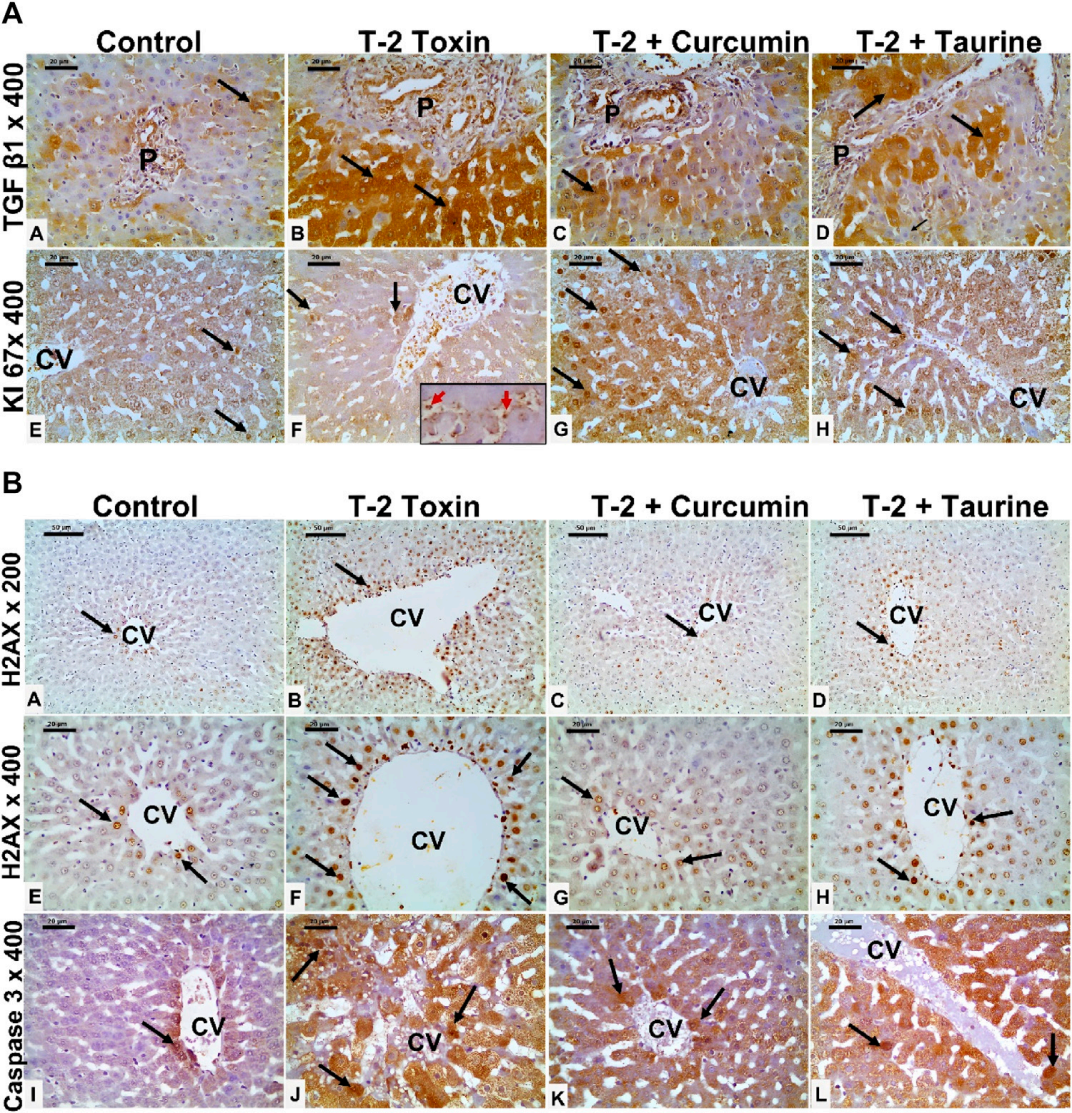
**(A)** Photomicrographs of liver tissue sections immunohistochemically stained for (anti-TGF-β1, and KI 67) from all groups. TGF-β1 showing minimal cytoplasmic immunoreaction (black arrow) in hepatocytes mainly at portal area of the control group (A), which is markedly upregulated in the T-2 toxin group (B). However, marked reduction TGF-β1immunoreactive hepatocytes (black arrow) in toxic group treated with curcumin treatment (C) and to less extent in toxic group treated with taurine (D) compared to toxic group. KI-67 positive immunoreactive cells (black arrow) showing marked decrease in T-2 toxin group with increased sinusoidal Kupffer cells immunoreactive (red arrow inside insert) (F) compared to control group (E). Positive immune reactive cells markedly increased in toxic group treated with curcumin (G) and moderately increased in toxic group treated with taurine (H) compared to toxic group. (Magnifications: Anti- TGF-β1, and KI 67 at 400, scale bar; 20 μm). **(B)** Photomicrographs of liver tissue sections immunohistochemically stained for (anti- H2AX, and active caspase 3) from all groups. Liver tissue sections immunohistochemically stained for Anti-phospho-Histone (H2AX) showing few nuclear positive hepatocytes (black arrow) around the central vein (CV) in control group (A,E). The number nuclear-positive hepatocytes markedly increased in the T-2 toxin group (B,F). However, the positive nuclear hepatocytes are markedly decreased in toxic group treated with curcumin (C,G) and moderately reduced in toxic group treated with taurine (D,H) compared to toxic group. Active caspase immunostaining showing marked increase in the number of immunoreactive hepatocytes (black arrow) in T-2 toxin (J) compared to control (I). The number of positive immune reactive hepatocytes markedly decreased in toxic group treated with curcumin (K) and moderately decreased in toxic group treated with taurine (L). (Magnifications: Anti- H2AX at 200 (A–D) and 400(E–H) with scale bar; 50 and 20 μm respectively and active caspase3 at 400 (I–L) with scale bar; 20 μm).

Collagen fibers deposition in liver tissue were evaluated by histochemical staining by Masson trichrome and Sirius red demonstrated a significant increase in liver fibrosis (*p* < 0.05) mainly around the portal area in T-2 toxin group comparing to control. Interestingly significant reduction (*p* < 0.05) in liver fibrosis mainly at the portal area was observed after curcumin was treated than taurine treated group compared to T-2 toxin group **(** Figures 2A, B **)**. PAS-stained magenta purple glycogen granules in hepatocytes, control group showed large amount of glycogen that is significantly reduced (*p* < 0.05) in T-2 toxin group. Following curcumin treatment hepatocytes content of glycogen is significantly increased more than with taurine in comparison to T-2 toxin group **(** Figures 2, 3C **)**.

#### 3.2.3 Immunohistochemical (anti- TGFβ-1, KI67, H2AX and active caspase3) results

**FIGURE 4:**
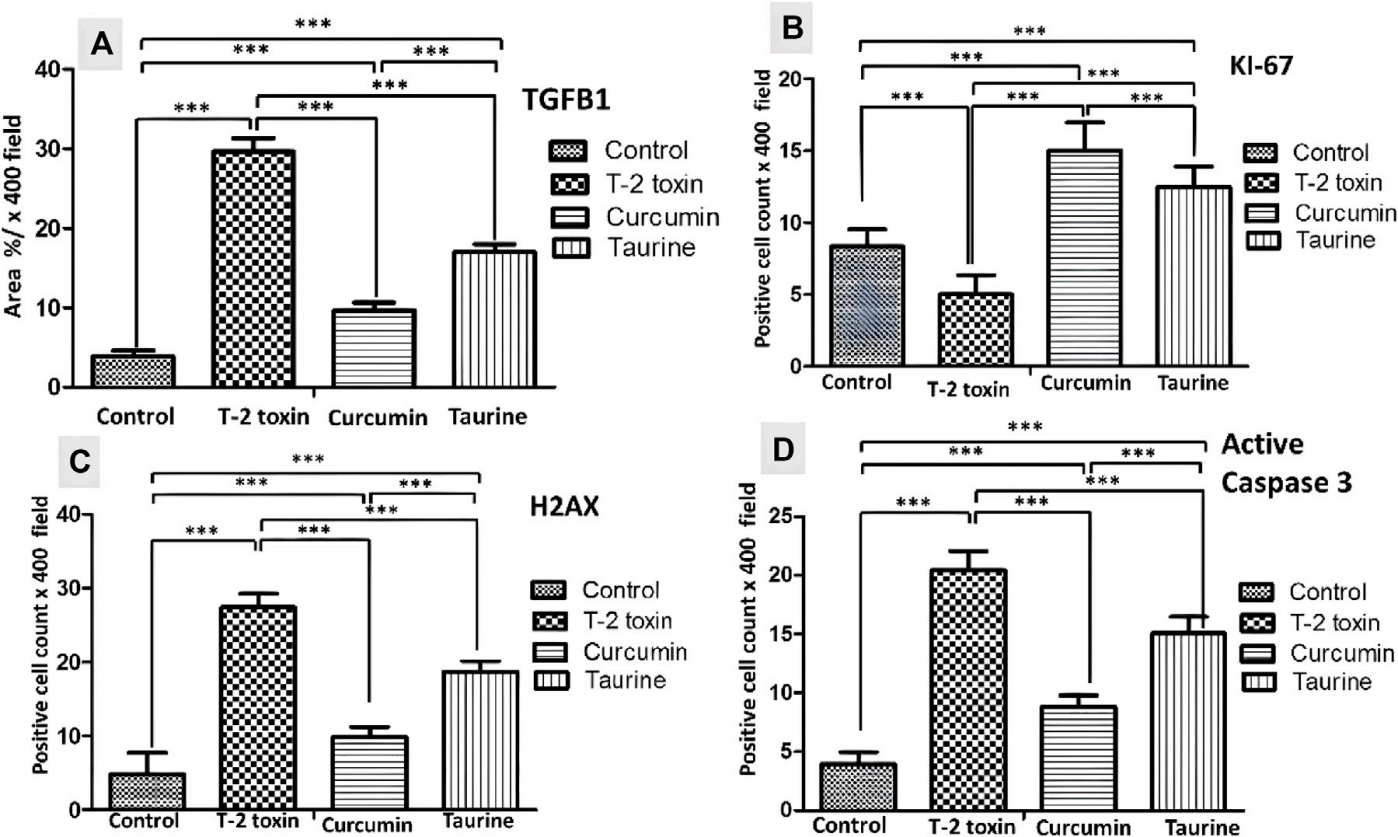
Collected Graphs for immunohistochemically morphometry statistical analysis of Area percentage of anti- TGF-β1 **(A)** and the number of positive KI 67 **(B)** H2AX **(C)** and Active caspase 3 hepatocytes **(D)**. Data are expressed as mean ± SD. ***P = significant difference less than 0.05, analysis of different liver tissue sections from all other groups.

Liver tissue expression of TGFβ-1 as a key regulator in fibrosis cascade was immunohistochemistry evaluated, expressed, and analyzed in **(** Figures 3A, 4A). Significant upregulation (*p* < 0.05) of TGFβ-1 immune-expression in periportal hepatocytes compared to control. Hepatocytes expression of TGFβ-1 was significantly downregulated (*p* < 0.05) because of curcumin treatment than taurine in comparison to T-2 toxin.

The regenerative capacity of liver tissue assessment by KI-67 as proliferating marker stain hepatocytes nuclei. KI-67 positive hepatocytes are significantly increased (*p* < 0.05) following curcumin more than taurine compared T-2 toxin group **(** Figures 3A, 4B **)**.

H2AX as a marker for double stranded DNA damage appeared as nuclear hepatocyte staining, showed a significant increase (*p* < 0.05) of H2AX positive hepatocytes in T- 2 toxin compared to the control group. Interestingly, positive hepatocytes count is significantly decreased following curcumin administration than taurine in comparison to T-2 toxin group (Figures 3B, 4C).

Also, Hepatocytes apoptosis, assessed by active caspase 3 as an apoptotic marker showed a significant increase (*p* < 0.05) of active caspase positive hepatocytes in T- 2 toxin compared to the control group. Interestingly, positive hepatocytes count is significantly decreased following curcumin administration than taurine in comparison to T-2 toxin group (Figures 3B, 4D).

### 3.3 Molecular modeling and docking analysis

#### 3.3.1 Molecular modeling of curcumin and taurine

**FIGURE 5:**
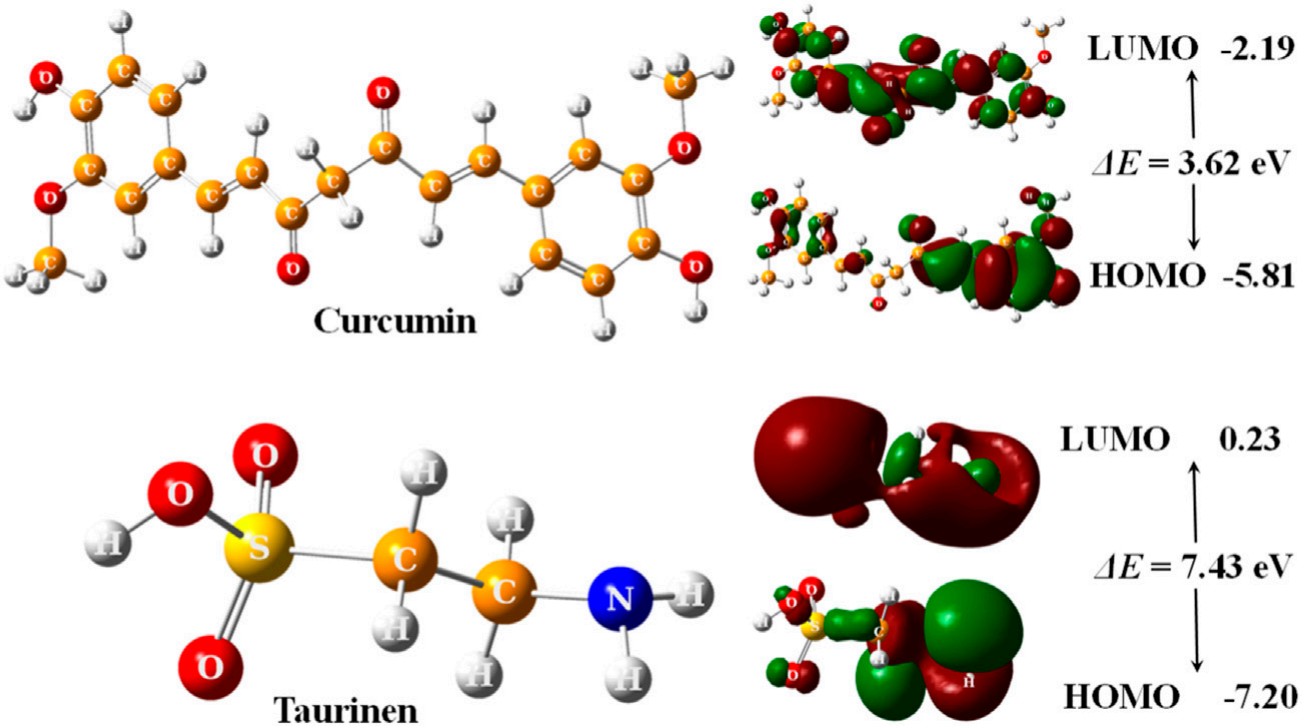
3D optimized structures of curcumin and taurine with their HOMO and LUMO molecular orbitals, calculated at B3LYP/6311g (d, p) level.

The optimized structures of curcumin and taurine with their highest occupied molecular orbital (HOMO) and lowest unoccupied molecular orbital (LUMO) are shown in Figure 5.

According to (Figure 5); Table 5, both structures were non-planar with delocalized electrons distribution in the HOMO and LUMO molecular orbitals. However, the energy gap (ΔE) of curcumin is small compared with taurine. Chemical tests were performed to determine the reactivity of the two compounds descriptors were estimated based on the EHOMO and ELUMO molecular orbitals, namely, ionization potential (IP), electron affinity (EA), chemical hardness (*η*), softness (*σ*), electronic chemical potential (*μ*), electronegativity (χ), electrophilicity index (*ω*), and nucleophilicity index (Nu), ∆E represents an essential parameter for compound activity, where small ∆E value corresponding for the reactive molecule. Table 5.

**TABLE 5:** Calculated EHOMO, ELUMO, ionization potential (IP), electron affinity (EA), energy gap (ΔE), Chemical hardness (*η*), Softness (*σ*), chemical potentials (*μ*), electronegativity (χ), electrophilicity index (*ω*), and nucleophilicity (Nu), by eV unit, of curcumin and taurine.

Model	EHOMO	ELUMO	IP	EA	∆E	η	σ	μ	χ	ω	Nu
Curcumin	−5.81	−2.19	5.81	2.19	3.62	1.81	0.55	−4.00	4.00	4.41	0.23
Taurine	−7.20	0.23	7.20	−0.23	7.43	3.71	0.27	−3.48	3.48	1.63	0.61

#### 3.3.2 Estimation of docking energy (ED) and H bonds interaction between substrate models and the active pocket residues of DNA

**FIGURE 6:**
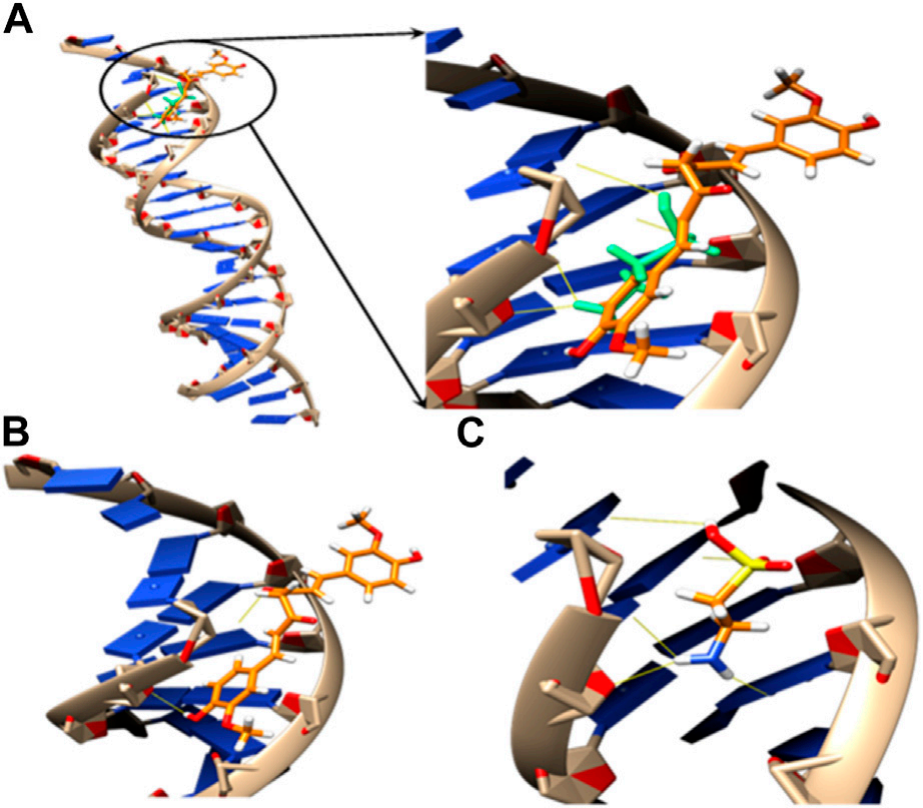
3D models of **(A)** superposed docking taurine (light green) and curcumin (orange), binding orientation with H-bonds in yellow of **(B)** taurine and **(C)** curcumin in the active pocket of DNA.

The relative orientations of taurine and curcumin are shown in (Figure 6); Table 6. Docking results showed dominant hydrophilic interactions in ligand/DNA complex. Taurine had electrostatic interaction with A: DG3, A: DG4, A: DA5, A: DA6, A:Da7, B: DT18, B:DC19, B:Dc20 nucleotides of DNA. Curcumin formed a hydrogen bond with s and showed hydrophobic interaction due to the presence of phenyl and methyl groups in its structure. The superimposed structure of the ligands in the DNA binding pocket revealed that both models accommodated the site where curcumin occupied a larger area because it has higher surface area than taurine. According to docking score, the calculated binding energies of curcumin and taurine are −37.59 kcal/mol and - 20.51 kcal/mol respectively. Table 6.

**TABLE 6:** Docking energy (ED) and H bonds interaction between substrate models and the active pocket residues of DNA.

Model	E_D_ [kJ/mol]	Donor	Acceptor	D. A dist., Å	Donor	H.A dist., Å
Curcumin	−37.59	DG 4004 N2	O5	3.23	H22	2.57
		O4	DT 5018 O2	4.39	H14	3.45
Taurine	−20.51	DG 4004 N2	O2	3.16	H22	2.25
		N1	DA 4006 N3	3.24	H5	2.26
		N1	DC 5019 O2	3.28	H6	2.56
		N1	DC 5019 O4	3.31	H6	2.43
		O1	DC 5020 O2	4.04	H7	3.48

## 4 Discussion

Right now, global mycotoxin contamination of food chain became a great disaster because of recent changes in climate and inadequate storage conditions, mostly affecting low-income countries. As a result, investigating the underlying cellular and molecular mechanism of the T-2 toxin as a common highly toxic fungal pollution of man and animals’ food is a stepway to evaluate its effects on biological tissues and the possible role of antioxidants agents. Mycotoxin induced liver toxicity is a medical hazards as the liver is a major metabolic organ (Ruan et al., 2022).

It was established that T-2 toxins provoked tissue damage via oxidative stress cytotoxicity and genotoxicity (Chaudhari et al., 2009; Adhikari et al., 2017; Yin et al., 2020) by producing reactive oxygen species (ROS) and downregulation of antioxidant enzymes (Janik et al., 2021). When ROS are excessive, they can damage intracellular biomolecules, such as lipids, proteins, DNA, mitochondria, and endoplasmic reticulum, which can result in cell death because of superoxide radical anions, hydroxyl radicals, hydrogen peroxide, and singlet oxygen (Schieber and Chandel, 2014). Antioxidants, such as glutathione (GSH), superoxide dismutase (SOD), and catalase (CAT), neutralize ROS rapidly under physiological conditions (Chandel, 2018). Curcumin and taurine as strong antioxidant were assessed in this study.

Curcumin, a common spice extracted in a pure crystalline form from Curcuma longa L. rhizomes (Turmeric) (Goel et al., 2008), is classified as a safe food and drug without any known side effects by the US Food and Drug Administration (Ammon and Wahl, 1991).

Liver enzymes measurement is used to describe liver functions, the data listed in Table 1 showed that, the level of liver enzymes ALT, AST and ALP were increased significantly by using T-2 toxin, this indicates the atrocious effect of the toxin on liver functions, but by using curcumin as a natural treatment, it observed a decrease in the level of enzymes. In addition, taurine treatment also exhibited good efficacy in decreasing liver enzymes.

These results were confirmed by a histological study where T-2 toxin-induced hepatic histopathological injury, including distorted lobular architecture, hepatocytes vacuolation, degeneration, ballooning, dilated blood sinusoids with increased Kupffer cell and massively dilated and congestion portal area with inflammatory cells infiltration (Liu et al., 2019), decreased hepatocytes glycogen and increased peri-portal fibrosis (Liu et al., 2021).

The curcumin ameliorated these toxic effects, and taurine treatment effectively restored the normal hepatic histological structure except for some blood congestion and cellular infiltration in taurine-treated groups. Curcumin and taurine upregulated the ability of hepatocytes for glycogen storage, which was abolished with the T-2 toxin. Also, curcumin and taurine ameliorated the hepatic fibrosis induced by T-2 toxin-induced liver fibrosis at the portal area (Ammon and Wahl, 1991; Damiano et al., 2021). Taurine also ameliorated fibrosis, although to a lesser extent than curcumin. These results were supported by other studies, which proved the role of curcumin and taurine in hepatic protection and liver injury progression through amelioration of oxidative stress (El–Houseini et al., 2017; Uzunhisarcikli and Aslanturk, 2019; Baliou et al., 2021; Shi et al., 2021; Younis et al., 2021).

Fibrosis assessment either histochemical by Masson trichrome and Sirius red or by immunohistochemistry anti-TGFβ1 marker as fibrotic marker indicated significant T-2 induced periportal. However, this curcumin and taurine showed significant amelioration of fibrosis. These anti-fibrotic activity results of curcumin and taurine are in concurrent with other studies on hepatic toxicity animal models induced liver fibrosis were treated with curcumin and taurine showed downregulated expression of TGFβ1 (Chen et al., 2018; Eshaghian et al., 2018; Yao et al., 2021; Mahmoudi et al., 2022).

The crosstalk between oxidative stress and DNA damage could be due to over production of ROS that damages proteins, lipids, and nucleic acids (Peluso et al., 2020; Cheng et al., 2021). DNA double-strand damage marker (anti-histone H2AX or H2AX) showed a significant decrease in immune reactive nuclei in both curcumin and taurine-treated groups, respectively. The anti-DNA damage effect of curcumin was proved in other studies on humans exposed to arsenic toxins (Roy et al., 2011; Maji et al., 2020), also taurine proven to attenuate DNA damage in different *in vivo* and *invitro* studies (Husain and Mahmood, 2020; Lai et al., 2022). This was associated with an increase in the proliferative and regenerative capacity of hepatocytes as evidenced by increased Ki67 positive nuclei in liver tissue following either curcumin or taurine therapy. It is proved that the reduction in the number or function of lobules and in hepato-toxicity leads to hepatic regeneration. It provoked the regenerative effects of curcumin and taurine in liver tissue in a certain study (Gozeneli et al., 2018).

Apoptosis (programmed cell death) is a vital physiological cell death process called can be brought on by noxious stimuli. T-2 toxin was previously reported induces cell death in thymus, spleen, and liver tissues, especially the liver. T-2 toxin decreased cell activity and enhanced apoptosis in chicken hepatocytes (Yin et al., 2020). In addition, the mitochondrial ROS-caspase apoptosis pathway was established as the mechanism through which T-2 toxin causes liver tissue damage in rabbits (Liu et al., 2021). So the antiapoptotic activity od curcumin and taurine could be attributed to their antioxidant effect.

T-2 toxin’s cytotoxicity has been found to be mediated by oxidative stress. Oxidative stress can cause cell death by activating the mitochondrial-caspase apoptosis pathway, as mitochondria are ROS’s most susceptible targets. ROS could induce mitochondrial membrane damage, lower its potential, and release the apoptotic factor. Cytochrome C (cytC) from mitochondria to cytoplasm (Tait and Green, 2010). Cytoplasmic cytC forms apoptotic bodies with apaf-1 and caspase-9, activating caspase-3. Apoptosis occurs eventually (Liu et al., 2021).

From the present study, it is clear that both total lipids and total cholesterol were increased by induction of T2 toxin; therefore, by treatment with taurine, the level of them decreased significantly, but curcumin exhibited a fabulous effect more than taurine in decreasing total lipid and cholesterol. This study agrees with other studies that found that mycotoxin levels are linked to hypercholesterolemia and has a negative impact on liver cholesterol metabolism via cholesterol production (Anyanwu et al., 2006; Martins, 2015; Grewal and Buechler, 2022). In human and animal plasma, mycotoxins have been found to attach to lipoproteins and cause hypercholesterolemia (Martins, 2015). Intestinal transport of cholesterol in lipoproteins such as chylomicrons and chylomicron remnants, as well as cholesterol delivery to the liver, are all impacts of a cholesterol diet (Lo et al., 2008; Lo and Coschigano, 2020). The liver synthesizes very low-density lipoprotein (VLDL). It transports cholesterol to peripheral tissues (Choi and Ginsberg, 2011), other studies inconsistent with our study in the effect of T2 toxin on the level of cholesterol, such that, the reduction in the concentration of serum cholesterol is most likely due to the inhibition of cholesterol biosynthesis and the reduction in the concentration of serum triglycerides might be associated with impaired lipid transport which causes from T-2 toxin, as suggested by (Anyanwu et al., 2006; Martins, 2015).

TBARS, which is used to measure lipid peroxidation, is considered as a lipid indicator peroxidation parameter. TBARS is widely used as a marker of lipid peroxidation and its high level reflects oxidative stress (Gaschler and Stockwell, 2017). T-2 toxin is suggested to enhance oxygen radical production, overloading the oxygen radical scavenging system, and causing cell injury, according to the previous results, it was found that injection of rats with T2 toxin according to the protocol led to an increase in TBARS and this ensures the role of T2 toxin in raising lipid peroxidation, however, treatment with taurine results as good treatment in decreasing TBARS as a lipid peroxidation parameter, curcumin as a natural treatment showed its effect in decreasing TBARS more than taurine, so, we can consider that curcumin is used as antioxidant treatment and have remarkable power as an antioxidant, other studies showed dual responses of mycotoxins were obtained, lower values of TBARS were found in exposure to slight increase thereafter, high value by using the highest dose (Kövesi et al., 2020). This result supports our previous explanation that T2 toxin increase the level of oxygen-free radical formation in the liver, leading to an increased TBARS level.

Reduced glutathione (GSH) is an important intracellular and extracellular antioxidant that controls signaling processes, detoxifies some xenobiotics and heavy metals and performs a variety of other functions. Cysteine, glutamic acid, and glycine make up this tripeptide. GSH concentrations in intracellular and whole blood are in the millimolar range, whereas plasma concentrations are in the micromolar range and account for around 0.4 percent of total blood GSH (Zinellu et al., 2008; Chwatko and Bald, 2009; Persichilli et al., 2011; Richie Jr et al., 2011; Wang et al., 2012). The erythrocytes contain practically all of the reduced glutathione (GSH) in the blood, and it becomes unstable when the red cells are exposed to medicines (Waggiallah and Alzohairy, 2011), where serum glutathione levels are used as an indicator for oxidative toxicants in the organs, and it was discovered that nucleophilic GSH conjugates a number of potentially toxic electrophilic xenobiotics (such as certain carcinogens), which is an important defense mechanism against certain compounds like drugs and carcinogens (Dasari et al., 2017). From the present study, it is clear that both total glutathione and blood GSH level were increased by inducing T2 toxin, some studies reveal that induction of T2 toxin decrease the production of GSH by downregulation the gene responsible of production of GSH, the inability of mycotoxin-affected cells to adequately generate glutathione, as well as impaired performance of glutathione-producing enzymes, might result in glutathione depletion on a long-term basis (Zazuli et al., 2018). However, another study discovered that inducing T2 toxin boosted GSH levels, which were associated with increased enzyme activity. This is consistent with Balogh et alearlier.'s findings (Balogh et al., 2007), the activation of GPx in the presence of its co-substrate can be explained. T-2 toxicity was quickly detected in blood plasma, which had dramatically elevated GSH concentration and GPx activity throughout the trial (Fernye et al., 2018).

According to the analyses, the induction of T2 toxin resulted in significantly higher mean values of AFU and TNF-Alpha than the control group. Hepatic diseases including hepatotoxicity, cirrhosis, early hepatic malignancy, and hepatitis have been linked to elevated serum AFU levels (Shuang et al., 2018). As a result, rising AFU due to T-2 toxin induction could be attributable to hepatotoxicity. The serum level of AFU was found to be helpful as a diagnostic index for primary hepatic cancer (Stefaniuk et al., 2010). Still, AFU in the current study was decreased in T2 and T3 treated with curcumin and taurine, respectively. These substances were considered hepatocellular protective as an antioxidative agent (El-Agamy, 2010). The treatment of increased serum TNF–Alpha with T-2 toxin increased the mean value compared to the control group. Still, treatment with curcumin resulted in a drop in the mean value compared to the T-2 toxin group.

TNF’s primary function is to regulate immune cells; however, TNF-Alpha also causes cell apoptosis by producing inflammation and preventing carcinogenesis and viral replication. As a result, the deregulation of TNF- Alpha production has been linked to several human illnesses, including cancer (Walczak, 2011). TNF-Alpha can be considered crucial for liver injury in T-2 toxin in the experimental rat (Dong et al., 2016). Trichothecene mycotoxins’ initial aim is leukocytes (Huang et al., 2007; Polak-Śliwińska and Paszczyk, 2021), demonstrating the effect of T-2 toxin on cytokine production by macrophages could help in understanding the mechanism by which mycotoxin compromises the immune system. Trichothecene also stimulates pro-inflammatory cytokine and chemokine production in mononuclear phagocytes by a mechanism known as the ribotoxic stress response, which involves the activation of numerous intracellular signaling cascades, according to Zhou et al. (2005). After that, treatment with taurine exhibited reduced levels of both tumor markers, but the reduction by treatment with curcumin is more than taurine. These results show that curcumin exhibited a more remarkable anti-inflammatory effect than taurine.

This study noticed a reduction in hemoglobin (Hb) levels due to the induction of the T-2 toxin. Hemoglobin and hematocrit are important clinical indicators of the T-2 toxin poisoning effect on red blood cells. These results agree with the result obtained by Manish Adhikari et al. (2017), who reported that the trichothecene mycotoxins destroy the hematopoietic system of bone marrow. These results showed the ability of the T-2 toxin to damage red blood cells membrane and hemolysis of red blood cells after exposure to the T-2 toxin (Nayakwadi et al., 2020).

Molecular modeling and docking analysis of molecular orbitals showed that both structures were non-planar with delocalized electron distribution in the HOMO and LUMO molecular orbitals. However, curcumin’s energy gap (ΔE) is small compared with taurine. This suggested that curcumin might be a more reactive compound. The reactivity of the two compounds was estimated based on the EHOMO and ELUMO molecular orbitals. From the results, it was founded that curcumin has a smaller ∆E value and thus would be more reactive than taurine. The reactivity trend from the hardness/softness point of view for curcumin follows the same direction, which agrees with the hard–soft–acid–base (HSAB) approximation rule. Hard acids like to interact with hard bases, while soft acids prefer to interact with soft bottoms. Biological systems such as enzymes, DNA, etc. are soft and interact easily with benign molecules. According to these parameters, curcumin is expected to be more reactive than taurine as a protective agent.

Docking was performed to investigate the binding of curcumin and taurine into the target DNA model. To elucidate unexplained experimental findings such as restoring damaged DNA function results from toxic compounds such as T2-toxin. Curcumin formed hydrogen bonds with s and showed hydrophobic interaction due to the presence of phenyl and methyl groups in its structure. The superimposed structure of the ligands in the DNA binding pocket revealed that both models accommodated the site where curcumin occupied a more extensive area because it has a higher surface area than taurine. According to the docking score and curcumin’s calculated binding energies, both substrates formed H-bonds with the surrounding DNA bases to stabilize their interaction. The high binding energy of curcumin suggested a high binding affinity to DNA and thus explains its high potential to cure DNA damage compared to taurine. Finally, these findings suggested that curcumin exerted a protective effect. Furthermore, it considers a more gorgeous treatment for repairing DNA damage than taurine. These results agree with biochemical and histological measurements in this study.

## 5 Conclusion

In conclusion, this study has described the oxidative stress-induced effect of T-2 toxin on hepatocytes structure and function through biochemicals, histopathological, immunohistochemistry and theoretical assays. The results showed a significant effect of T-2 toxin on serum levels of antioxidants and other parameters as GsH, HB, Ht, AFU and TNF-Alpha and liver tissue degeneration, inflammation and vascular congestion, fibrosis and glycogen depletion and hepatocytes DNA damage and apoptosis. Furthermore, curcumin and taurine were found to be extraordinary antioxidant natural products that influence the hepatotoxicity of T-2 toxin, ameliorate all the histopathological parameters, and improve liver antioxidant function with a preference for curcumin over taurine. Added to our knowledge T-2 Toxin is one of the essential trichothecene mycotoxins occurring naturally in agricultural products involved in severe field cases of human Toxicoses and farm animals.

## Data Availability

The original contributions presented in the study are included in the article/Supplementary Material, further inquiries can be directed to the corresponding author.
